# Supplementary material for: Dietary Patterns Associated with Diabetes in an Older Population from Southern Italy Using an Unsupervised Learning Approach
Source: Sensors (Basel). 2022 Mar 11;22(6):2193. doi: 10.3390/s22062193 (PMC8949084; doi:10.3390/s22062193)
Supplement: Supplementary file 1 [file sensors-22-02193-s001.zip › sensors-1611277-supplementary.pdf]

sensors

Dietary Patterns Associated with Diabetes in an Older Population from Southern Italy Using an Unsupervised Learning Approach

Rossella Tatoli [1], Luisa Lampignano [1,*], Ilaria Bortone [1,*], Rossella Donghia [1], Fabio Castellana [1], Roberta Zupo [1], Sarah Tirelli [1], Sara De Nucci [1], Annamaria Sila [1], Annalidia Natuzzi [1], Madia Lozupone [2], Chiara Griseta [1], Sabrina Sciarra [1], Simona Aresta [1], Giovanni De Pergola [3], Paolo Sorino [4], Domenico Lofù [4], Francesco Panza [2], Tommaso Di Noia [4] and Rodolfo Sardone [1]

[1] Unit of Data Sciences and Technology Innovation for Population Health, National Institute of Gastroenterology "Saverio de Bellis", Research Hospital, 70013 Bari, Italy; rossella.tatoli@irccsdebellis.it (R.T.); rossydonghia@irccsdebellis.it (R.D.); fabio.castellana@irccsdebellis.it (F.C.); zuporoberta@gmail.com (R.Z.); sarah.tirelli@irccsdebellis.it (S.T.); sara.denucci@irccsdebellis.it (S.D.N.); annamaria.sila@irccsdebellis.it (A.S.); annalidia.natuzzi@irccsdebellis.it (A.N.); chiara.griseta@irccsdebellis.it (C.G.); sabrinasciarra@hotmail.it (S.S.); arestasimo@gmail.com (S.A.); rodolfo.sardone@irccsdebellis.it (R.S.)

[2] Neurodegenerative Disease Unit, Department of Basic Medicine, Neuroscience, and Sense Organs, University of Bari Aldo Moro, 11, 70125 Bari, Italy; madia.lozupone@gmail.com (M.L.); f_panza@hotmail.com (F.P.)

[3] Unit of Geriatrics and Internal Medicine, National Institute of Gastroenterology "Saverio de Bellis", Research Hospital, 70013 Bari, Italy; gdepergola@libero.it

[4] Department of Electrical and Information Engineering, Polytechnic of Bari, 70125 Bari, Italy; paolo.sorino@irccsdebellis.it (P.S.); domenico.lofu@poliba.it (D.L.); tommaso.dinoia@poliba.it (T.D.N.)

* Correspondence: luisa.lampignano@irccsdebellis.it (L.L.); ilariabortone@gmail.com (I.B.)

Table S1. Concordance of the single foods in the questionnaire and the food grouping used in the analyses.

	Final Food Groups	Single Foods from Questionnaire
1	DAIRY	Latte intero (*whole-fat milk*), Scamorza-Caciottina fresca-Stracchino-Fontina (semi-seasoned italian cheese), Bel Paese-Gorgonzola (italian blue cheese), Provolone-Caciocavallo (seasoned italian cheese), Grana-Parmigiano, Svizzero (*swiss cheese*), Pecorino-Vacchino (*goat cheese, cow cheese*), Formaggino, (*cheese spread*), Mozzarella (*mozzarella cheese*), Gelato (*ice cream*), Yogurt
2	LOW FAT DAIRY	Latte scremato–parzialmente scremato (*skimmed and semi-skimmed milk*), Ricotta (*cottage cheese*)
3	EGGS	Uova (*eggs)*
4	WHITE MEAT	Pollo (*chicken*), Coniglio (*rabbit*)
5	RED MEAT	Vitello (*veal*), Cavallo (*horse*), Maiale (*Pork*), Fegato (*liver*), Agnello (*lamb*)
6	PROCESSED MEAT	Salsiccia fresca (*fresh sausages*), Prosciutto crudo (*raw ham*), Mortadella (a tipical italian cured meat),Prosciutto cotto (ham), Salame (*salami*)
7	FISH	Sogliola-Orata-Dentice-Spigola-Cernia (*sole, sea bream, snapper, sea bass, grouper*), Merluzzo-Razza-Palombo (*codfish, stingray, dogfish*), Triglia-Cefalo-Sgombro (*goatfish, mullet, mackerel*), Acciughe-Sarde (*anchovies, sardines*), Tonno sott'olio (*tuna in oil*)
8	SEAFOOD/SHELLFISH	Polpo-Seppie-Calamari-Gamberi (*octopus, cuttlefish, squid, prawns)*, Cozze-Altri frutti di mare (*mussels, other seafoods*)
9	LEAFY VEGETABLES	Spinaci (*spinach*), Bietole-Cicorie (*chard, chicory*), Insalata (*salad*)
10	FRUITING VEGETABLES	Pomodori (*tomatoes*), Zucchine-Melanzane (*zucchini, eggplants*), Peperoni (*peppers*), Carciofi (*artichokes*) Cetrioli-cocomeri (*cucumbers*)

11	ROOT VEGETABLES	Carote (*carrots*)
12	OTHER VEGETABLES	Minestrone (*vegetable soup*), Cavoli–Cavolfiori-Cime di Rape-Rape (*cabbage, cauliflower, broccoli, green turnips*), Finocchi- Sedano (*fennels, celery)*
13	LEGUMES	Ceci - Lenticchie–Fagioli (*chickpeas, lentils, beans*), Piselli (*peas*), Fagiolini (*green beans*), Fave con Verdura (*broad beans with vegetables*)
14	POTATOES	Patate (*potatoes*)
15	FRUITS	Arance-Mandarini-Pompelmi (*oranges, tangerines, grapefruits*), Pesche (*peaches*), Fichi (*figs*), Albicocche (*apricots*), Uva (*grapes*), Anguria (*watermelon*), Melone giallo (*melon*), Mele-Pere (*apples, pears*), Kiwi, Ciliege (cherries), Banane
16	NUTS	Frutta secca (*nuts*)
17	GRAINS	Pane (*bread*), Pasta asciutta (*pasta*), Riso o risotti (*rice or risotti*), Pastina o riso in brodo (*pasta or rice in broth*)
18	OLIVES AND VEGETABLE OIL	Olive da tavola (*olives*), Olio di oliva (*olive oil*)
19	COOKING EDIBLE FATS	Olio di semi (*seed oil*), Olio di oliva per frittura (*olive oil for frying*), Olio di semi per frittura (*seeds oil for frying*), Olio di oliva per cucinare (*olive oil for cooking*), Olio di semi per cucinare (*seeds oil for cooking*), Burro (*butter*), Margarina (*margarine*), Burro per frittura (*butter for frying*), Margarina per frittura (*margarine for frying*), Burro per cucinare (*butter for cooking*), Margarina per cucinare (*margarine for cooking*)
20	SWEETS	Caramelle (*sweets*), Cioccolata (*chocolate*), Pasticceria (*pastries*), Biscotti–Paste secche (*cookies, biscuits*)
21	SUGARY	Zucchero (*sugar*), Frutta sciroppata (*fruit in syrup*)
22	JUICES	Succhi di frutta (*fruit juice*)
23	CALORIC DRINKS	Coca Cola–Aranciata–Chinotto (*coke, orange juice, chinotto*)
24	READY TO EAT DISH	Pizza, Focaccia (a tipical Apulian bakery product)
25	COFFEE	Caffè (*coffee*), Caffè d'orzo (*barley coffee*)
26	WINE	Vino (*wine*)
27	BEER	Birra (*beer*)
28	SPIRITS	Liquore (*liquor*)
29	WATER	Acqua (*water*)
